# Evaluating reasoning models for therapy recommendations in gastrointestinal stromal tumors: expert and LLM-based evaluations of OpenAI o1 and DeepSeek-R1

**DOI:** 10.1007/s00432-026-06489-7

**Published:** 2026-05-18

**Authors:** Melissa Harbrücker, Franka Menge, Arshia Taebi, Dominik Nörenberg, Tobias Speer, Christoph Reißfelder, Peter Hohenberger, Jens Jakob, Chengpeng Li, Cui Yang

**Affiliations:** 1 Department of Surgery, Medical Faculty Mannheim, University Medical Center Mannheim, University of Heidelberg, Mannheim, Germany; 2 DKFZ-Hector Cancer Institute, Medical Faculty Mannheim, Heidelberg University, Mannheim, Germany; 3 Department of Radiology and Nuclear Medicine, Medical Faculty Mannheim, University Medical Center Mannheim, Heidelberg University, Mannheim, Germany; 4 Independent Researcher, Ludwigshafen Am Rhein, Germany; 5 Division of Surgical Oncology & Thoracic Surgery, Department of Surgery, Medical Faculty Mannheim, University of Heidelberg, Mannheim, Germany; 6 Key Laboratory of Carcinogenesis and Translational Research (Ministry of Education/Beijing), Sarcoma Center, Peking University Cancer Hospital & Institute, Beijing, China

**Keywords:** Gastrointestinal stromal tumor, Large language model, Artificial intelligence, Multidisciplinary, Decision-making, Human-in-the-loop

## Abstract

**Purpose:**

This study aims to evaluate two advanced reasoning LLMs in generating treatment recommendations for real-world gastrointestinal stromal tumor (GIST) cases and assess their concordance with multidisciplinary team (MDT) decisions at a certified tertiary sarcoma center.

**Methods:**

Sixty-five real-world GIST cases from a tertiary sarcoma center were used to compare two advanced reasoning models—OpenAI o1 and DeepSeek-R1. Recommendations were generated using a multi-expert prompting strategy with current clinical guidelines as context. Five sarcoma specialists and an independent LLM (Mistral AI) evaluated alignment with MDT decisions and guideline concordance.

**Results:**

OpenAI o1 achieved higher concordance with MDT decisions than DeepSeek-R1 (76.9% vs. 53.8%, *p* < 0.001) and more recommendations aligned with either MDT or guidelines (80.0% vs. 63.1%, *p* = 0.005). Inter-rater reliability among human evaluators was excellent (ICC = 0.929). The LLM judge’s evaluations showed moderate agreement with human assessments (κ = 0.647). OpenAI o1 responses were significantly longer than those of DeepSeek-R1 and MDT records.

**Conclusions:**

OpenAI o1 outperformed DeepSeek-R1 in generating clinically relevant GIST therapy recommendations. The study highlights the feasibility of using LLMs both as decision support tools and as evaluators (“LLM-as-a-judge”) in oncology, while emphasizing the need for expert oversight in clinical deployment.

**Supplementary Information:**

The online version contains supplementary material available at 10.1007/s00432-026-06489-7.

## Introduction

Soft tissue sarcomas (STS) account for less than 1% of the adult malignancies (Stiller et al. 2013). Among these, gastrointestinal stromal tumors (GIST) are the most common mesenchymal tumors of the gastrointestinal tract and account for approximately 20–25% of all STSs, representing a rare cancer entity (Joensuu et al. 2013). The management of GISTs poses significant challenges in decision making in real-world settings, since there is a need for the integration of clinical information, molecular biology findings and multimodal imaging as well as a complex mutation-specific treatment adjustment. Thus, multidisciplinary team (MDT) involvement is a necessity in the therapeutic decision-making process for GIST (Bonvalot et al. 2019). Findings from the German PROSa trial highlighted the critical role of MDTs in GIST management, which reported that the utilization rate of multidisciplinary tumor boards (MTBs) in accredited sarcoma centers vs other hospitals is associated with an odds ratio (OR) of 5.39 (95% CI 3.28–8.85) (Eichler et al. 2021). However, a recent ring trial evaluating the concordance of sarcoma MDTs at certified sarcoma centers revealed significant variabilities, underscoring the complexity of the sarcoma treatment strategy (Roohani et al. 2025).

Typically, MDT recommendations are developed within a compact sequence of cases which often might limit in-depth discussions. Within this, clinicians are confronted with a flood of information from the patient history, pathology, radiology, molecular biology, and genetics due to the rapidly evolving possibilities of next-generation sequencing (NGS), new clinical trial results and brand-new biomarkers data deducted from histology-agnostic panels (Horak et al. 2021). There is an increasing requirement for streamlining of MDTs to increase effectiveness (Merker et al. 2023).

To address this problem, large language models (LLMs)—as an artificial intelligence (AI) tool—have been proposed as a decision support tool in oncology. According to our prior work (Li et al. 2024, 2025), GPT models could answer STS questions in alignment with the German S3 guideline, especially when retrieval augmented generation (RAG) approach was implemented. Given their ability to analyze large volumes of medical data as required for MDTs (Yamamoto et al. 2024), LLMs may have to potential as a supporting tool for MDTs. Despite this potential, there are only a few studies investigating the performance of LLMs as assistants in MDT discussions (Hamamoto et al. 2022; Sorin et al. 2023; Schmidl et al. 2024; Aghamaliyev et al. 2024). Prior studies have evaluated ChatGPT’s performance in gastrointestinal and sarcoma tumor boards, revealing heterogeneous accuracy levels (Aghamaliyev et al. 2024; Ammo et al. 2025).

This study aims to assess the utility of LLMs in providing recommendations for real-world GIST cases. Specially, we evaluated the concordance between recommendations generated by two reasoning models and decisions made by MDTs in a certified tertiary sarcoma center.

## Methods

**Fig. 1:**
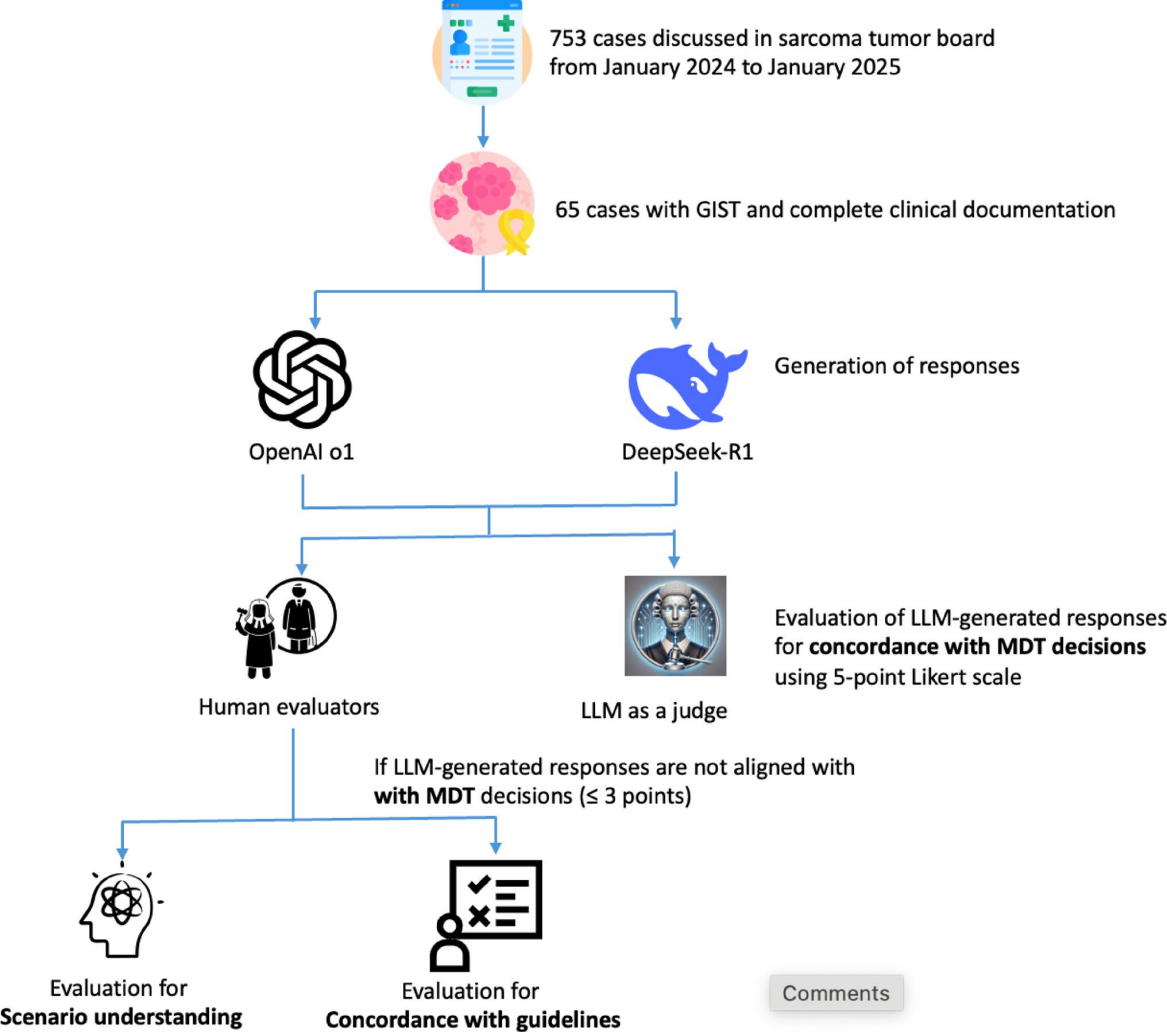
General workflow of the study. Free icons from Flaticon (https://www.flaticon.com/) and images generated by DALL·E were used. GIST, gastrointestinal stromal tumor; LLM, large language model; MDT, multidisciplinary team

This study was a retrospective, single-center analysis using data from 753 cases which were discussed at the interdisciplinary sarcoma tumor board of University Hospital Mannheim of University Heidelberg between January 2024 and January 2025. Of these, 65 cases met the inclusion criteria, including diagnosis or suspicion of GISTs, complete clinical documentation and clearly documented MDT recommendations. The study was approved by the University Faculty Ethics Committee and Institutional Review Board at University Hospital Mannheim of Heidelberg University (#2024-837) and registered in the German Clinical Trials Register (DRKS00034227 |http://www.germanctr.de/) on September 27th.2024. The study followed the TRIPOD-LLM guidelines for AI-based research reports (Gallifant et al. 2025) (TRIPOD checklist please see supplementary file 1). The workflow of the study is illustrated in Fig. 1

### Data and data pre-processing

Following data were extracted from the electronic health records (HER) and translated into English for analysis:

- *Patient status* age, sex, Eastern Cooperative Oncology Group (ECOG) performance status, comorbidities and wills of patients.
- *Diagnostics* endoscopy findings, radiological reports and pathological results.
- *Oncological course* date of first diagnosis, recurrent/metastasis cites, prior and current treatments, response to therapy.
- MDT query and decisions.

To protect patients’ privacy, all data were anonymized rigorously before they were entered into LLMs. Not only were direct identifiers such as names and record numbers removed, but their ages were also provided as 5 years-range (e.g. 55–60 years) instead of exact ages. All specific dates were replaced by only month and year information. Cases with missing data were excluded from the analysis. The data were stored in an excel spreadsheet, to which only authorized research personnel hat access.

### LLM selection

We chose two advanced reasoning models: OpenAI o1 was included as a state-of-the-art closed-source LLM and DeepSeek-R1 as a rising star open-source model. We did not select OpenAI o3 because only a limited “mini” version was available at the time of the study.

### Multi-expert prompting framework

Multi-expert prompting has been shown to increase the performance of LLMs (Long et al. 2024). To simulate a real-world MDT, we developed a prompting strategy instructing the models to take over multiple specialist roles (e.g. surgical oncologist, medical oncologist, radiologist and pathologist). The prompt also provided instructions guiding the behavior of each “specialist”. The models simulated a discussion through the voices of different specialists, their responses were aggregated into the final consensus in a pre-defined structured format (pleases see supplementary file 2).

### Generation of responses

In this approach, we provided the multi-expert prompt as system prompt and the case via the chat interfaces on February 20th, 2025. Additionally, the German S3 clinical guidelines for adult soft tissue sarcomas (English version) (German Cancer Society 2022) was provided to the LLMs. For OpenAI o1, we uploaded the guidelines as a PDF document via the web interface (www.chatgpt.com). Since the DeepSeek-R1 interface (www.deepseek.com) did not support file upload, we accessed DeepSeek-R1 through the Chatbox platform (chatboxai.app).

### Human evaluation

A panel of five human evaluators (two attending sarcoma surgeons, one attending oncologist specialized in sarcoma, and two surgical residents from a sarcoma team) independently rated the final consensus of LLM-generated recommendations. The evaluators were blinded to the sources of the recommendations and evaluated the concordance with the MDT decisions using a 5-point Likert scale described in the evaluation scale, with five indicating completely consistent and one not consistent at all. All evaluators were instructed that only the content should be evaluated, and linguistic formulation (wording) is irrelevant.

If the discrepancy between the scores of raters was more than one, the raters engaged in a discussion to review their ratings to come to a consensus. The medians of the scores were employed for further statistical analysis.

If a recommendation received a score of ≤ 3 and thus diverged from the MDT decision, two sarcoma attending surgeons (CPL and CY) independently performed two evaluations of the LLM outputs. In the first evaluation, they rated whether the models “understood” the case scenario correctly using a 5-point Likert scale (1 = completely misunderstood, 5 = completely understood). In the second evaluation, they assessed the degree to which the recommendations aligned with the German S3 guidelines using the same scale (1 = not aligned at all, 5 = totally aligned). For the evaluation scales, please see supplementary file 3.

### LLM as a judge

We employed a third LLM, Mistral AI as a judge (Zheng et al. 2023) of concordance between each LLM’s recommendation and the actual MDT decisions. To improve our workflow efficiency, we used the Mistral AI Application Programming Interfaces (API) for programmatic interactions with mistral-large-latest. The model was prompt with the evaluation scale used for human evaluation, the actual MDT decision and LLM-generated recommendation. For the prompts used, please see supplementary file 4.

### Statistical analysis

Statistical analyses were performed using SPSS Statistics (IBM Corp. Released 2023. IBM SPSS Statistics for MacOS, Version 29.0.2.0 Armonk, NY: IBM Corp). Figures were drawn using GraphPad Prism (GraphPad Prism version 10.3.1 for MacOS, GraphPad Software, Boston, Massachusetts USA, www.graphpad.com) and Microsoft® PowerPoint for MacOS (version 19.95.1). The normality of continuous variables was evaluated using the Shapiro–Wilk test. An intraclass correlation coefficient (ICC) is used to measure the reliability of ratings among five human raters. Cohen’s weighted kappa statistic was used to quantify the consistency of the scores between the human raters and LLM-judge. Group-wise comparisons were conducted using the Wilcoxon rank-sum test, Wilcoxon signed-rank test, Chi-squared test or paired t-test, as appropriate and all tests were two-sided. The threshold for statistical significance was set at *p* < 0.05.

## Results

In total, 65 GIST cases were included in the final analysis. In most cases, patients were between 41 and 80 years old at the time of MDT discussion, and the sex distribution was approximately equal (47.7% female, 52.3% male). 15 (23.1%) cases were in the stage of primary diagnosis, 28 (43.1%) in postoperative setting and 22 (33.8%) had recurrent or metastatic disease. The most common primary tumor site was the stomach (41 cases, 63.1%), followed by small intestines (21 cases, 27.7%) and colon/rectum (3 cases, 4.6%) (Table 1).

**Table 1:** Case descriptions

Category	Subcategory	N (%)
Age range	21–30	2 (3.1%)
	31–40	1 (1.5%)
	41–50	10 (15.4%)
	51–60	16 (24.6%)
	61–70	22 (33.8%)
	71–80	11 (16.9%)
	81–90	3 (4.6%)
Sex	Male	34 (52.3%)
	Female	31 (47.7%)
Interval from first diagnosis to discussion	Median (range), months	3.8 (0.5–322.5)
	Primary diagnosis	15 (23.1%)
	Postoperative care	28 (43.1%)
Disease status	Recurrent/metastatic disease	22 (33.8%)
	Liver	7 (10.7%)
	Peritoneum	5 (7.7%)
	Multiple sites	10 (15.4%)
	Stomach	41 (63.1%)
	Duodenum	9 (9.2%)
Primary tumor site	Jejunum/ileum	12 (18.5%)
	Colorectal	3 (4.6%)
	Unknown	3 (4.6%)

### Models’ alignment with MDT and guideline-concordant decisions—human evaluation

**Fig. 2:**
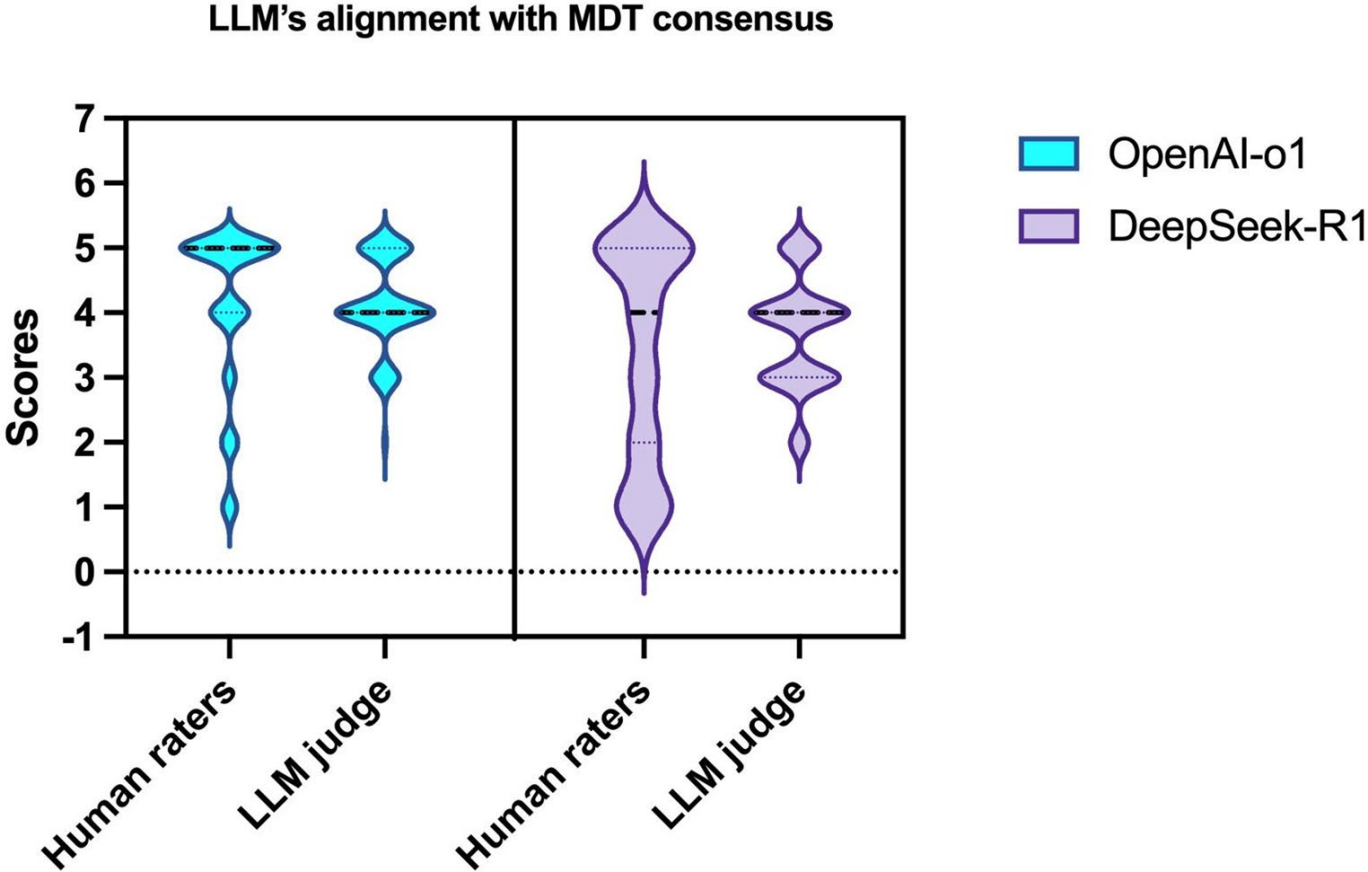
Violin plot showing the distribution of scores for alignment of the LLMs with the MDT consensus, as rated by the human raters and the LLM judge, respectively. *LLM*, large language model

**Fig. 3:**
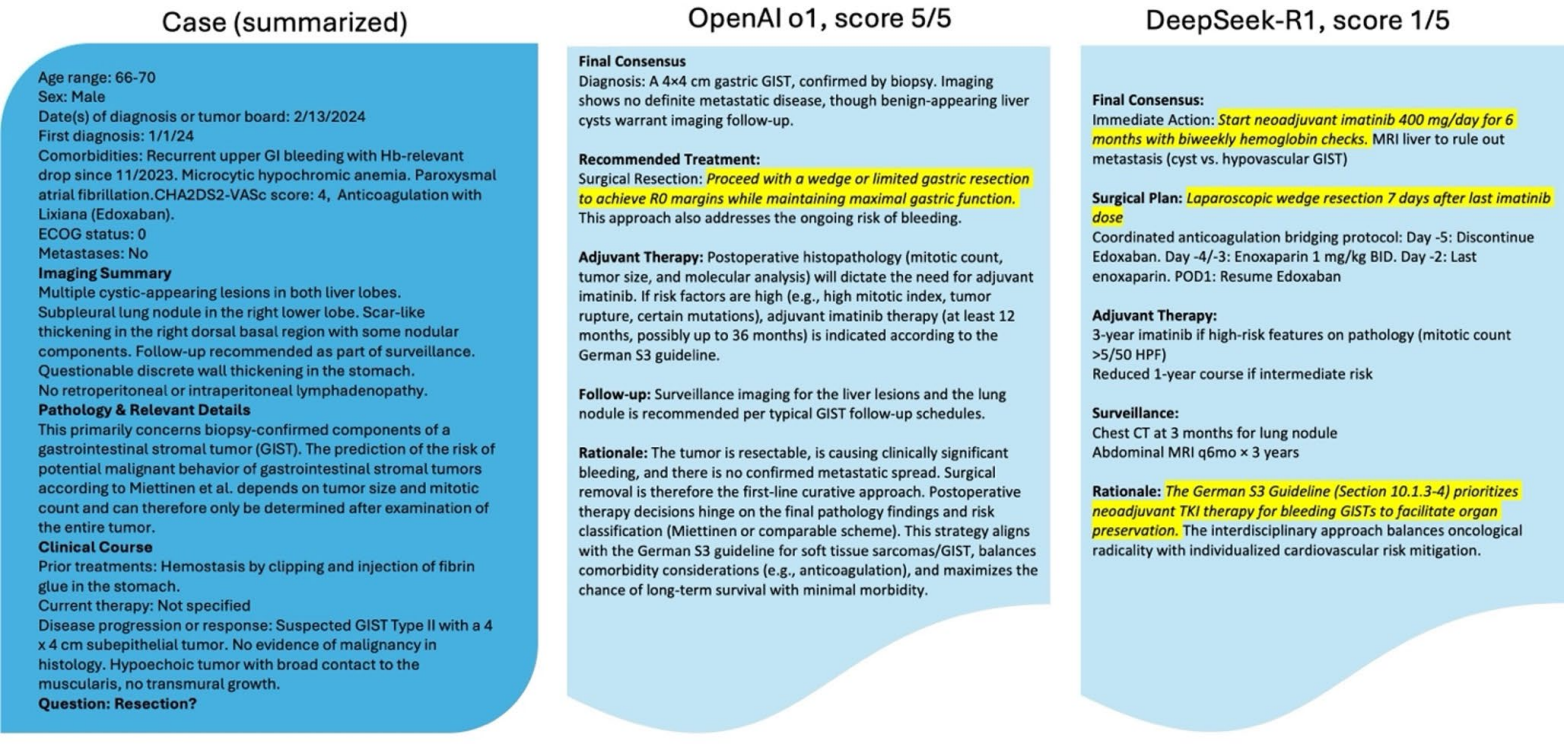
An example of LLM-generated recommendations and their scores rated by human evaluators. In the response of DeepSeek-R1, a hallucination was observed: neither 10.1.3 nor 10.1.4 suggested neoadjuvant TKI therapy for bleeding GISTs. These two sections were about “Adjuvant drug therapy of localized, R0 resected GIST” and “Clinical management of metastatic GIST”. *GIST*, gastrointestinal stromal tumor; *TKI*, tyrosine kinase inhibitor

OpenAI o1 demonstrated significantly greater alignment with the MDT decisions than DeepSeek-R1 (OpenAI o1: median = 5; range: 1–5; DeepSeek-R1: median = 4; range: 1–5; *p* < 0.001) (Table 2 and Fig. 2). For OpenAI o1, 50 recommendations (76.9%) achieved more than three points, indicating that their clinical content was completely or mostly consistent with the MDT recommendations, which is significantly higher than that for DeepSeek-R1 (35, 53.8%, *p* < 0.001). Among those, 35 (53.8%) recommendations generated by OpenAI o1 showed complete alignment (rating = 5), compared to 27 (41.5%) generated by DeepSeek-R1 (p = 0.044). An example case and according LLM-generated recommendations (final consensus) are presented in Fig. 3. For all cases with recommendations (final consensus) generated by OpenAI o1 and DeepSeek-R1, please see supplementary file 5.

**Table 2:** Alignment between LLMs-generated recommendations and multidisciplinary team decisions evaluated by human raters and an LLM-judge

	OpenAI o1	Deepseek-R1	*p* value
Human raters	5 (1–5)	4 (1–5)	< 0.001
LLM judge	4 (2–5)	4 (2–5)	< 0.001

*LLM* large language model

Intramodal analysis showed that, compared with less challenging questions about the diagnosis of primary disease or postoperative therapy, the performance of OpenAI o1 did not significantly decline when answering questions related to recurrent or metastatic tumors (all *p*-values > 0.05). In contrast, Deepseek-R1 exhibited clear subgroup stratification. Performance in the recurrent/metastatic setting differed significantly from both primary disease (*p* = 0.005) and postoperative care (*p* = 0.003), while no difference was observed between the latter two (*p* = 0.927).

In subgroup analyses, no significant differences in alignment were observed between OpenAI o1 and Deepseek-R1 for primary disease and postoperative care–related queries (all *p* > 0.05). However, for recurrent/metastatic scenarios, OpenAI o1 achieved higher alignment scores than Deepseek-R1 (median 4 [range 1–5] vs 2 (Stiller et al. 2013; Joensuu et al. 2013; Bonvalot et al. 2019; Eichler et al. 2021; Roohani et al. 2025), *p* < 0.001), indicating a divergence in performance under more complex clinical conditions. (Table 3).

**Table 3:** Subgroup comparison of LLMs-generated recommendations evaluated by human raters

	OpenAI o1	Deepseek-R1	*p* value
Primary disease	5 (2–5)	5 (1–5)	0.219
Postoperative care	5 (1–5)	5 (1–5)	0.263
Recurrent/metastatic disease	4 (1–5)	2 (1–5)	< 0.001

*LLM* large language model

While OpenAI o1 exhibited 15 outputs (23.1%) rated ≤ 3 indicating misalignment with MDT decisions, DeepSeek-R1 displayed a significantly higher count of 30 outputs (46.2%) rated ≤ 3. In the subsequent evaluation of recommendations rated ≤ 3, the median score for scenario understanding of OpenAI o1 was 5 (range 1–5). Two responses were not evaluable for guidelines alignment because there was no corresponding recommendation in the S3 Guidelines. For the remaining 13 responses, the median score evaluated for alignment with the guidelines was 3 (range: 1–5) and only two responses (15.4%) were fully aligned with the guidelines. In contrast, the median score for scenario understanding of DeepSeek-R1 was 4.25 (range 1–5). The median score of the responses’ alignment with the S3 guidelines was 3 (range: 1–5) and six responses (20%) were fully aligned with the guidelines. The differences between models were not significant.

Totally, 52 responses (80.0%) generated by OpenAI o1 were either aligned with the MDT decisions or with the current guidelines, and by DeepSeek-R1 41 responses (63.1%). The difference was significant (*p* = 0.005).

### Inter-rater reliability among human evaluators

Evaluation of model outputs’ alignment with the MDT decisions was conducted independently by five clinical experts. Inter-rater agreement was excellent, with an ICC = 0.929 (95% CI [0.909, 0.946], *p* < 0.001), confirming a high degree of consistency among human evaluators. Evaluation of the unaligned outputs (ratings ≤ 3) to determine scenario understanding and their alignment with the S3 guidelines, Cohen’s weighted kappa statistic showed a statistically significant reliability of 0.975 (95% CI [0.955,0.995], *p* < 0.001), indicating an almost perfect level of agreement among the two raters.

### Model alignment with MDT and guideline-concordant decisions—LLM-as-a-judge

OpenAI o1 demonstrated higher alignment with the MDT decisions (median = 4; range: 2–5) than DeepSeek-R1 (median = 4; range: 2–5; *p* < 0.001) according to the ratings of the LLM judge (Fig. 2). For OpenAI o1, 19 responses (29.2%) achieved 5 points and were thus completely aligned the MDT decisions. 11 responses (16.9%) generated by DeepSeek-R1 showed complete alignment. While OpenAI o1 exhibited 12 outputs (18.5%) rated ≤ 3 indicating misalignment with MDT decisions, R1 displayed a significantly higher count of 28 outputs (43.1%) rated ≤ 3.

### Agreement between human evaluators and LLM-as-a-judge

To assess whether LLMs could emulate human evaluators in rating MDT alignment, we compared the judgments of an independent third LLM (Mistral AI) against the aggregated ratings of five clinical experts. Agreement between the human raters (median scores) and the LLM judge was moderate, with a Cohen’s quadratically weighted kappa of 0.647 (SE = 0.043, 95% CI [0.562, 0.732], *p* < 0.001) (McHugh 2012).

### Output verbosity

A comparison of character counts revealed significantly longer outputs from OpenAI o1 (mean = 1359, SD = 408) compared to DeepSeek-R1 (mean = 697 SD = 146) character counts (*p* < 0.001). Both models provided significantly longer outputs than that of the MDT discussion (mean = 79, SD = 58) (all *p* values < 0.001).

## Discussion

To the best of our knowledge, this is the first study to evaluate two advanced reasoning models, OpenAI o1 and DeepSeek-R1, for their decision-making capabilities to generate recommendations for real-world GIST cases. Overall, OpenAI o1 demonstrated higher concordance with MDT decisions compared to DeepSeek-R1, regardless of whether the responses were evaluated by human raters or by an independent LLM (Mistral AI). In the subset of outputs that diverged from MDT decisions, both models showed reasonable levels of scenario understanding, but insufficient guideline alignment. Our findings also indicate that Deepseek-R1 performs less well in more complex clinical scenarios. This suggests that Deepseek-R1 is less capable of handling clinically challenging questions, with a clear decline in performance as case complexity increases. In contrast, OpenAI o1 maintained stable alignment across all subgroups, without deterioration in more advanced disease contexts.

Our findings support that reasoning LLMs, especially OpenAI o1, might have the potentials to serve as assistants in clinical decision-making in the oncology and thus improve MDT workflow. Previous data in the literature are controversial. In the proof-of-concept study of Sorin et al., ChatGPT-3.5 generated similar recommendations to the tumor board decisions in 7 out of 10 breast cancer cases (Sorin et al. 2023), while the retrospective analysis of Aghamaliyev et al. showed that 83% of ChatGPT-3.5 generated recommendations were concordant with MDT decisions for overall treatment strategy for gastrointestinal tumors but only 65% for the exact treatment strategy (Aghamaliyev et al. 2024). Schmidl et al. demonstrated that both ChatGPT-4o and 4.0 cannot tailor therapy recommendations and made wrong treatment options in complex recurrent/metastatic head and neck squamous cell carcinomas cases (Schmidl et al. 2024). Ammo et al. provided the first insight into using ChatGPT-4o in handling sarcoma-related scenarios and could only show moderate effectiveness (Ammo et al. 2025). When using an enhanced system, the concordance between LLM suggestions and expert recommendations was reported to be 94.7% within molecular tumor boards (Lammert et al. 2024). Several factors could explain why the findings are heterogenous. Firstly, the selection of cases varied across studies. Unlike prior studies using fictional or simplified patient vignettes (Ammo et al. 2025; Lammert et al. 2024), we used unfiltered, current real-world cases. Real-world data are usually messy free text and thus challenging for LLM interpretation (Wiest et al. 2024). Furthermore, our cases reflect the real-world setting from primary diagnosis to complex treatment strategies. In contrast, Schmidl et al. selected complex recurrent/metastatic diseases, Aghamaliyev et al. only included first-time diagnoses and cases presented post-surgery and Lammert et al. focused on molecularly targeted treatment options. Secondly, different models or versions of models were used. While previous versions of ChatGPT were used in the studies mentioned above, we utilized two advanced reasoning models, which stand out for their abilities to deal with complex problems. By breaking down complex questions into a series of logical steps, incomplete or incorrect answers can be reduced (Wei et al. 2022). OpenAI o1 was shown to outperform GPT-4 in clinical decision-making tasks (Gunes et al. 2025). Thirdly, since oncology is a rapid evolving field, with ever-expanding medical literature and guidelines being updated regularly, we included current cases from January 2024 to January 2025 to reduce the risk of comparing older MDT decisions with LLM-generated responses based on current knowledge base and guidelines. Lastly, we simply presented the whole guidelines in the context, which provided the LLMs a comprehensive knowledge base to draw from when formulating recommendations. RAG was implemented to enhance the LLMs by providing selected relevant context in the MEREDITH system (Lammert et al. 2024). In other previous studies, such extra knowledge base was not provided.

A critical aspect of evaluations involving MDT decisions is adherence to clinical guidelines. Clinical guidelines represent the foundational practices that should be universally applied (the “floor”), rather than the highest possible standard of care (the “ceiling”) currently achievable in medical practice, because clinical guideline might be overtaken by rapidly evolving medical science (Guerra-Farfan et al. 2023). This was demonstrated in previous studies, in which variability in clinical decision making varied widely even among expert centers (Roohani et al. 2025; Guerra-Farfan et al. 2023; Rothermundt et al. 2023). Accordingly, MDT decisions do not always align with existing guidelines. In some cases, clinicians incorporate the most current evidence from the literature, which may not yet be reflected in formal recommendations of clinical guidelines. Some LLM-generated outputs, while diverging from local MDT preferences, still fell within acceptable guideline-based reasoning (Hamamoto et al. 2022; Matsuoka et al. 2024). To become a reliable assistant, LLMs must at least reach the “floor standard” and be adherent to current guidelines. OpenAI o1 demonstrated a higher reliability by generating 80% recommendations which were aligned with either MDT decision or guidelines, which might qualify it as a reliable assistant.

The tendency of GPT-based models to produce more detailed and structured is consistent with prior work (Li et al. 2024; Ammo et al. 2025; Matsuoka et al. 2024), and was reinforced by the character count analysis in our study showing significantly longer outputs generated by LLMs than MDT decisions. Since medical experts usually have very tight schedules in the tumor board discussion, these richer responses may improve the quality and efficiency of MDT documentation. However, this verbosity—while beneficial for transparency—may also pose usability challenges. In fast-paced MDT environments, long and verbose outputs may impede quick interpretation, echoing the concerns raised by Merker et al. about information overload and the need for streamlining case discussions (Merker et al. 2023).

While DeepSeek-R1 was shown to excel in tasks such as mathematics and coding (DeepSeek-AI et al. 2025), its performance was inferior in our specific setting. Its architecture including features such as the Mixture-of-Experts model, is optimized for enhancing efficiency, which might limit the depth in reasoning for complex clinical scenarios. Proprietary models like OpenAI o1 might benefit from access to high-quality clinical datasets and fine-tuning on medical literature. However, DeepSeek-R1’s open-source nature offers some advantages such as transparency, customizable retraining and cost-effectiveness. While commercial models are superior in clinical reasoning, open-source alternatives enable improvement through community-driven development, which is important for long-term implementation in healthcare systems.

Human evaluation remains essential for assessing the performance of LLMs, although it is usually time consuming. Our findings suggest that the LLM judge moderate level of alignment with human evaluators and the use of LLMs as independent, objective evaluators (“LLM-as-a-judge”) could be promising. The overall conclusions drawn—regarding MDT decision alignment and guideline adherence—were comparable whether ratings originated from humans or from the LLM. Although the absolute rating scores differed, the results of comparisons were consistent of those of human evaluators. While LLMs cannot fully substitute for human judgment, these findings suggest a potential role for LLMs as judges in combination with human-in-the-loop evaluators (Fast et al. 2024).

This study adds to a growing body of evidence that LLMs, particularly OpenAI o1, can meaningfully augment MDT workflows. However, their integration must be approached cautiously. Ethical and regulatory concerns—including transparency, accountability, and equity of access—must be addressed before deployment at scale (Ong et al. 2024).

### Limitations and future directions

Our study has some limitations. Firstly, LLMs generated recommendations only based on text. In the real-world MDT, radiological images are usually demonstrated in addition to written reports. This is in particular advantageous when special questions such as tumor resectability or suspected tumor progression come up. Secondly, we provided the guideline along with the prompt by simply uploading it. This approach has the risk of overloading the LLM’s context window and it is cost intensive due to the huge amount of tokens. It is also difficult for the model to identify the most relevant guideline recommendations for a given scenario. However, this approach is easy to use and no programmatical knowledge is necessary, which fits the need of most physicians. Thirdly, we only included the current guidelines as guidance. Since clinical guidelines may not be up-to-date, further references such as literature research with high quality randomized trials on the research topic within an agentic approach should be considered (Lammert et al. 2024). Lastly, despite our structured prompting, LLMs remain black-box systems. Their reasoning pathways cannot yet be fully audited. The development of clinician-in-the-loop feedback systems and reinforcement learning methods will be vital for improving model accuracy and clinical integration. Although we employed a multi-expert prompting strategy, it does not fully replicate the interactive nature of a real-world MDT. In clinical practice, MDT decisions emerge from repeated exchanges among specialists and re-evaluation of imaging and pathology. In our design, the deliberative process occurs within a single model and a single inference context; we therefore cannot know with certainty how the simulated specialist opinions are internally resolved. Future studies should explore multi-round, agentic LLM workflows and compare these more directly with real MDT decision-making to provide a fairer evaluation.

## Conclusion

OpenAI o1 outperformed DeepSeek-R1 in generating recommendations for GIST cases that aligned with MDT decisions and clinical guidelines. The findings underscore the potential of reasoning models as assistant for multidisciplinary tumor board. Our findings further indicate that an LLM-based evaluator can provide judgments broadly similar with human raters, which could reduce the burden of expert review while preserving reliability. Nonetheless, given the complexity of oncology practice, LLMs must be deployed under close expert supervision. Future research should expand upon these results by exploring multi-center cohorts, involving multimodal models, investigating retrieval-augmented methods for enhancing guideline adherence, and refining human-in-the-loop feedback systems to ensure safe, efficient, and equitable integration of LLMs into cancer care.

## Supplementary Information

Below is the link to the electronic supplementary material.

Supplementary Material 1

Supplementary Material 2

Supplementary Material 3

Supplementary Material 4

Supplementary Material 5

## Data Availability

The anonymized clinical data are made available as supplementary files and will be published on heiDATA (https://heidata.uni-heidelberg.de/), an institutional repository for Open Research Data from Heidelberg University. Further data could be made available upon reasonable request to the corresponding authors.

## Code availability

The code will also be publicly available via: https://github.com/Chimik-IT/LLM4Tumor in form of an app for the command line interface (CLI). The app will make it easier for individuals not familiar with Python coding to get quick results for their projects.

## References

[CR1] Aghamaliyev U, Karimbayli J, Giessen-Jung C et al (2024) ChatGPT’s gastrointestinal tumor board tango: a limping dance partner? Eur J Cancer 205:114100. 10.1016/j.ejca.2024.114100 38729055 10.1016/j.ejca.2024.114100

[CR2] Ammo T, Guillaume VGJ, Hofmann UK et al (2025) Evaluating ChatGPT-4o as a decision support tool in multidisciplinary sarcoma tumor boards: heterogeneous performance across various specialties. Front Oncol 14:1526288. 10.3389/fonc.2024.1526288 39896191 10.3389/fonc.2024.1526288 PMC11782276

[CR3] Bonvalot S, Gaignard E, Stoeckle E et al (2019) Survival benefit of the surgical management of retroperitoneal sarcoma in a reference center: a nationwide study of the French Sarcoma Group from the NetSarc database. Ann Surg Oncol 26:2286–2293. 10.1245/s10434-019-07421-9 31065964 10.1245/s10434-019-07421-9

[CR5] Eichler M, Andreou D, Golcher H et al (2021) Utilization of interdisciplinary tumor boards for sarcoma care in Germany: results from the PROSa study. Oncol Res Treat 44:301–312. 10.1159/000516262 33887740 10.1159/000516262 PMC8220922

[CR6] Fast D, Adams LC, Busch F et al (2024) Autonomous medical evaluation for guideline adherence of large language models. Npj Digit Med 7:358. 10.1038/s41746-024-01356-6 39668168 10.1038/s41746-024-01356-6 PMC11638254

[CR7] Gallifant J, Afshar M, Ameen S et al (2025) The TRIPOD-LLM reporting guideline for studies using large language models. Nat Med 31:60–69. 10.1038/s41591-024-03425-5 39779929 10.1038/s41591-024-03425-5 PMC12104976

[CR8] German Cancer Society (2022) German Guideline Program in Oncology (German Cancer Society, German Cancer Aid, AWMF): Soft Tissue Sarcoma Long version 1.1, 2022, AWMF Registration Number: 032/044OL

[CR9] Guerra-Farfan E, Garcia-Sanchez Y, Jornet-Gibert M et al (2023) Clinical practice guidelines: the good, the bad, and the ugly. Injury 54:S26–S29. 10.1016/j.injury.2022.01.047 35135686 10.1016/j.injury.2022.01.047

[CR10] Gunes YC, Cesur T, Camur E et al (2025) Textual proficiency and visual deficiency: a comparative study of large language models and radiologists in MRI artifact detection and correction. Acad Radiol. 10.1016/j.acra.2025.01.004 39939230 10.1016/j.acra.2025.01.004

[CR4] Guo, D., Yang, D., Zhang, H. et al. DeepSeek-R1 incentivizes reasoning in LLMs through reinforcement learning. Nature 645, 633–638 (2025). 10.1038/s41586-025-09422-z 10.1038/s41586-025-09422-z PMC12443585 40962978

[CR11] Hamamoto R, Koyama T, Kouno N et al (2022) Introducing AI to the molecular tumor board: one direction toward the establishment of precision medicine using large-scale cancer clinical and biological information. Exp Hematol Oncol 11:82. 10.1186/s40164-022-00333-7 36316731 10.1186/s40164-022-00333-7 PMC9620610

[CR12] Horak P, Heining C, Kreutzfeldt S et al (2021) Comprehensive genomic and transcriptomic analysis for guiding therapeutic decisions in patients with rare cancers. Cancer Discov 11:2780–2795. 10.1158/2159-8290.CD-21-0126 34112699 10.1158/2159-8290.CD-21-0126

[CR13] Joensuu H, Hohenberger P, Corless CL (2013) Gastrointestinal stromal tumour. Lancet 382:973–983. 10.1016/S0140-6736(13)60106-3 23623056 10.1016/S0140-6736(13)60106-3

[CR14] Lammert J, Dreyer T, Mathes S et al (2024) Expert-guided large language models for clinical decision support in precision oncology. JCO Precis Oncol. 10.1200/PO-24-00478 39475661 10.1200/PO-24-00478

[CR15] Li C-P, Jakob J, Menge F et al (2024) Comparing ChatGPT-3.5 and ChatGPT-4’s alignments with the German evidence-based S3 guideline for adult soft tissue sarcoma. iScience. 10.1016/j.isci.2024.111493 39759026 10.1016/j.isci.2024.111493 PMC11699281

[CR16] Li C-P, Jia W-W, Chu Y et al (2025) Improving accuracy and source transparency in responses to soft tissue sarcoma queries using GPT-4o enhanced with German evidence-based guidelines. Oncol Res Treat. 10.1159/000544978 40024240 10.1159/000544978

[CR17] Long DX, Yen DN, Luu AT, et al (2024) Multi-expert prompting improves reliability, safety, and usefulness of large language models 10.48550/arXiv.2411.00492

[CR18] Matsuoka M, Onodera T, Fukuda R et al (2024) Evaluating the alignment of artificial intelligence‐generated recommendations with clinical guidelines focused on soft tissue tumors. J Surg Oncol. 10.1002/jso.27874 39233558 10.1002/jso.27874

[CR19] McHugh ML (2012) Interrater reliability: the kappa statistic. Biochem. 10.11613/BM.2012.031 PMC3900052 23092060

[CR20] Merker L, Conroy S, El-Wakeel H, Laurence N (2023) Streamlining the multi-disciplinary team meeting: the introduction of robust pre-preparation methods and its effect on the length of case discussions. JMDH 16:613–622. 10.2147/JMDH.S387174 36910017 10.2147/JMDH.S387174 PMC9993954

[CR21] Ong JCL, Chang SY-H, William W et al (2024) Ethical and regulatory challenges of large language models in medicine. The Lancet Digital Health 6:e428–e432. 10.1016/S2589-7500(24)00061-X 38658283 10.1016/S2589-7500(24)00061-X

[CR22] Roohani S, Handtke J, Hummedah K et al (2025) The sarcoma ring trial: a case-based analysis of inter-center agreement across 21 German-speaking sarcoma centers. J Cancer Res Clin Oncol 151:30. 10.1007/s00432-024-06063-z 39755880 10.1007/s00432-024-06063-z PMC11700044

[CR23] Rothermundt C, Andreou D, Blay J-Y et al (2023) Controversies in the management of patients with soft tissue sarcoma: recommendations of the Conference on State of Science in Sarcoma 2022. Eur J Cancer 180:158–179. 10.1016/j.ejca.2022.11.008 36599184 10.1016/j.ejca.2022.11.008

[CR24] Schmidl B, Hütten T, Pigorsch S et al (2024) Assessing the role of advanced artificial intelligence as a tool in multidisciplinary tumor board decision-making for primary head and neck cancer cases. Front Oncol 14:1353031. 10.3389/fonc.2024.1353031 38854718 10.3389/fonc.2024.1353031 PMC11157509

[CR25] Sorin V, Klang E, Sklair-Levy M et al (2023) Large language model (ChatGPT) as a support tool for breast tumor board. Npj Breast Cancer 9:44. 10.1038/s41523-023-00557-8 37253791 10.1038/s41523-023-00557-8 PMC10229606

[CR26] Stiller CA, Trama A, Serraino D et al (2013) Descriptive epidemiology of sarcomas in Europe: report from the RARECARE project. Eur J Cancer 49:684–695. 10.1016/j.ejca.2012.09.011 23079473 10.1016/j.ejca.2012.09.011

[CR27] Wei J, Wang X, Schuurmans D, et al (2022) Chain-of-thought prompting elicits reasoning in large language models. 10.48550/arXiv.2201.11903

[CR28] Wiest IC, Ferber D, Zhu J et al (2024) Privacy-preserving large language models for structured medical information retrieval. Npj Digit Med 7:257. 10.1038/s41746-024-01233-2 39304709 10.1038/s41746-024-01233-2 PMC11415382

[CR29] Yamamoto A, Koda M, Ogawa H et al (2024) Enhancing medical interview skills through AI-simulated patient interactions: nonrandomized controlled trial. JMIR Med Educ 10:e58753. 10.2196/58753 39312284 10.2196/58753 PMC11459107

[CR30] Zheng L, Chiang W-L, Sheng Y, et al (2023) Judging LLM-as-a-Judge with MT-Bench and Chatbot Arena 10.48550/arXiv.2306.05685

