# Supplementary material for: Evaluating reasoning models for therapy recommendations in gastrointestinal stromal tumors: expert and LLM-based evaluations of OpenAI o1 and DeepSeek-R1
Source: J Cancer Res Clin Oncol. 2026 May 18;152(7):136. doi: 10.1007/s00432-026-06489-7 (PMC13357472; doi:10.1007/s00432-026-06489-7)
Supplement: Supplementary file 3 — Supplementary Material 3 [file 432_2026_6489_MOESM3_ESM.docx]

Evaluation Scale: Consistency with the MDT Decision (GIST Therapy, including Palliative Context, (Re)Imaging, and Clinical Trials)

**Definition:**

This scale assesses how closely the LLM-generated recommendation aligns with the historical MDT decision for GIST therapy. Key factors include:

- Treatment intent (curative vs. palliative)

- Selection of TKI line and dosing (e.g., Imatinib, Sunitinib, Regorafenib, Ripretinib, Avapritinib)

- Timing/indication for surgery (if relevant)

- (Re)Imaging/restaging (intervals, modalities)

- Monitoring and supportive care

- Clinical trial recommendations (e.g., referral to a relevant trial if standard options are limited or if the MDT specifically recommended trial participation)

- Consideration of mutational/resistance profiles

Only the content is evaluated; linguistic formulation (wording) is irrelevant.

Rating Scale (1–5):

**5 – Completely Consistent**

- The LLM recommendation matches the MDT decision in all critical aspects for the specific disease stage and treatment goal (curative or palliative). This may include:

- Correct TKI line and dosing (e.g., Imatinib first-line for metastatic disease, adjuvant Imatinib post-surgery, Sunitinib second-line, etc.)

- Accurate surgical strategy (timing, indication), consistent with the MDT plan

- (Re)Imaging/restaging intervals align with the MDT recommendation (e.g., the same or highly similar schedule for CT/MRI)

- Consideration of mutational status (e.g., PDGFRA D842V)

- If a specific clinical trial was recommended by the MDT (due to lack of standard options or resistance concerns), the LLM acknowledges or matches that same trial referral

- Any minor differences are clinically negligible (e.g., minor wording or slight variations in a schedule that do not change the actual management).

**4 – Mostly Consistent**

The LLM recommendation mirrors the overall MDT strategy and intent (curative vs. palliative) but shows small discrepancies in some details, for example:

- Correct TKI choice and surgical indication, but slight variation in exact dosage or treatment duration

- Minor differences in (re)imaging frequency (e.g., recommending scans every 9–10 weeks instead of every 8 weeks) that are unlikely to significantly affect patient outcomes

- A clinical trial might be noted, but details differ slightly from the MDT’s suggestion (e.g., same phase study but different location), without fundamentally altering the intended approach

- The fundamental treatment approach and goal remain clearly preserved.

**3 – Moderately Consistent**

There is partial alignment with the MDT decision, but also notable deviations that may affect patient care, for example:

- The general TKI selection or line is correct, yet the dosage, therapy duration, or timing might be suboptimal or unclear

- The (re)imaging schedule deviates enough to potentially delay detection of progression or treatment response (e.g., significantly longer intervals than recommended)

- The approach to surgery or the overall intent (curative vs. palliative) is somewhat aligned but includes decisions that could pose risks or lead to less optimal outcomes

- These differences are substantial enough that they might alter clinical decision-making or quality of care.

**2 – Minimally Consistent**

- The LLM recommendation retains only a few core elements from the MDT plan, with most details significantly mismatched, for example:

- The recommended TKI might be correct in name, but placed in the wrong treatment line (e.g., second-line in a first-line scenario or vice versa)

- Critical discrepancies in (re)imaging (e.g., recommending minimal or no imaging in a scenario where close monitoring is essential)

- Important surgery-related decisions (timing, intent) are overlooked or incorrectly applied (e.g., proposing immediate surgery in an advanced, unresectable stage)

- The overall divergence is likely to compromise patient management compared to the MDT plan.

**1 – Not Consistent**

- The LLM recommendation is fundamentally at odds with the MDT decision, likely resulting in a completely different and potentially harmful treatment strategy, for example:

- Misalignment of intent: A curative approach is replaced by a palliative regimen (or vice versa), ignoring the actual disease stage

- Omission of an indicated TKI in metastatic disease or the use of a blatantly inappropriate TKI line (e.g., Sunitinib first-line for no valid reason)

- Neglecting crucial (re)imaging or follow-up strategies, risking failure to detect progression or evaluate treatment response

- Ignoring major mutational considerations (e.g., prescribing Imatinib in the presence of a known PDGFRA D842V mutation)

- The recommendation could drastically impact patient outcomes, indicating a near-total departure from the MDT plan.
