# Supplementary material for: Evaluating reasoning models for therapy recommendations in gastrointestinal stromal tumors: expert and LLM-based evaluations of OpenAI o1 and DeepSeek-R1
Source: J Cancer Res Clin Oncol. 2026 May 18;152(7):136. doi: 10.1007/s00432-026-06489-7 (PMC13357472; doi:10.1007/s00432-026-06489-7)
Supplement: Supplementary file 4 — Supplementary Material 4 [file 432_2026_6489_MOESM4_ESM.docx]

Please act as an impartial judge and evaluate the quality of the responses provided by two AI assistants to the user question displayed below. Your evaluation should assess the **consistency,** assesses how closely the answers generated by both AI assistants align with the reference statement in terms of overall strategy, therapeutic approach, and clinical intent.

**Evaluation Scale (1–5):**

**5 – Fully Consistent:**
The LLM recommendation reflects the MDT decision in all critical aspects. It follows the same treatment strategy and underlying clinical rationale, with only negligible differences (e.g., minor variations in wording) that do not alter the overall direction.

**4 – Largely Consistent:**
The recommendation is mostly aligned with the MDT decision, with only minor discrepancies in wording or minor details. The overall therapeutic approach and clinical reasoning remain essentially consistent with the MDT decision.

**3 – Moderately Consistent:**
There is a partial alignment between the LLM recommendation and the MDT decision. Some key components match, but notable differences in the therapeutic approach or emphasis may influence the clinical interpretation.

**2 – Minimally Consistent:**
The LLM recommendation shows only limited alignment with the MDT decision. While some elements are similar, significant differences in overall strategy or critical clinical details raise doubts about its validity.

**1 – Not Consistent:**
The LLM recommendation deviates significantly from the MDT decision. The therapeutic approach and clinical reasoning differ to an extent that could lead to different clinical outcomes.

You will be given a reference statement, assistant A’s answer, and assistant B’s answer. Avoid any position biases and ensure that the order in which the responses were presented does not influence your decision. Do not allow the length of the responses to influence your evaluation. Do not favor certain names of the assistants. Be as objective as possible. Output your final verdict by strictly following this format: Consistence of assistant A is [], consistence of assistant B is []. Explanation:"".

[The Start of Reference statement]

{answer_ref}

[The End of Reference statement]

[The Start of Assistant A’s Answer]

{answer_a}

[The End of Assistant A’s Answer]

[The Start of Assistant B’s Answer]

{answer_b}

[The End of Assistant B’s Answer]
